# Evaluating the Impact of Airborne Fine Particulate Matter and Heavy Metals on Oxidative Stress via Vitamin Supplementation

**DOI:** 10.3390/toxics12070465

**Published:** 2024-06-27

**Authors:** Eunji Lee, Seonmi Hong, Yong-Dae Kim, Dae-In Lee, Sang-Yong Eom

**Affiliations:** 1 Department of Preventive Medicine, College of Medicine, Chungbuk National University, Cheongju 28644, Republic of Korea; lej0312@korea.kr (E.L.); seonmi@chungbuk.ac.kr (S.H.); ydkim@chungbuk.ac.kr (Y.-D.K.); 2 Chungbuk Environmental Health Center, Chungbuk National University Hospital, Cheongju 28644, Republic of Korea; 3 Chungbuk Regional Cancer Center, Chungbuk National University Hospital, Cheongju 28644, Republic of Korea; 4 Cardiovascular Center, Korea University Guro Hospital, Korea University College of Medicine, Seoul 08308, Republic of Korea

**Keywords:** particulate matter, heavy metals, oxidative stress, malondialdehyde, antioxidants, vitamins

## Abstract

This cross-sectional study aimed to assess the interrelationships between PM_2.5_ exposure, heavy metal concentrations, and oxidative stress indicators, while evaluating the impact of antioxidant intake, such as vitamins, on these associations. PM_2.5_ exposure assessments were conducted using portable sensor-based monitors; biomarker analyses for heavy metals and oxidative stress were performed in 114 non-smoking adults. We observed that personal or ambient PM_2.5_ exposure levels were not associated with increased levels of heavy metals in blood and urine, nor with oxidative stress levels in urine. However, the concentrations of cadmium and lead in blood, and those of chromium and nickel in urine, were significantly associated with the urinary malondialdehyde (MDA) concentration. Additionally, increases in blood cadmium, urinary chromium, and nickel levels were significantly associated with higher urinary MDA concentrations in the non-vitamin-supplement group, but this trend was not observed in the regular vitamin supplement group. Our findings suggest that a regular intake of vitamin supplements might modulate the relationship between heavy metal exposure and oxidative stress, indicating potential protective effects against oxidative damage induced by PM_2.5_ and heavy metals. This study highlights the complexity of environmental pollutant exposure and its impact on human health, emphasizing the need for further research to elucidate the underlying mechanisms and explore potential protective strategies.

## 1. Introduction

Airborne fine particulate matter (PM_2.5_) is respirable dust with an aerodynamic diameter of less than 2.5 μm [1]. PM_2.5_ occurs naturally from forest fires, volcanic eruptions, and desert winds; it is also produced by human activities, in outputs such as automobile exhaust, factory soot, road dust, and cooking fumes [1,2]. In 2019, ambient particulate matter was responsible for 4.14 million premature deaths [3]. In addition, PM_2.5_ exposure causes a variety of chronic diseases in humans, including cardiovascular disease (CVD), stroke, asthma, and chronic obstructive pulmonary disease [1,2,3]. The International Agency for Research on Cancer has identified air pollution, including PM_2.5_, as a human carcinogen [4].

PM_2.5_ is composed of numerous chemical components, including heavy metals such as lead, cadmium, and arsenic, and polycyclic aromatic hydrocarbons, with its composition varying depending on the source [1,5]. Among the various components of fine particulate matter, heavy metals, including group 1 carcinogens like cadmium and arsenic, can seriously impact human health. Choi et al. analyzed the PM_10_ content and 15 types of metals in four cities in Korea and found that the average PM_10_ levels in Incheon and Seoul were 56.26 µg/m^3^ and 34.74 µg/m^3^, respectively. The average concentrations of the 15 metals were 12.8 µg/m³ and 7.7 µg/m^3^, respectively, accounting for approximately 22% of the PM_2.5_ composition [6]. Liu et al. reported that, during the non-heating period, PM_2.5_-bound lead and cadmium concentrations ranged from 5.41 to 44.68 ng/m^3^ and from 112.02 to 853.36 ng/m³, respectively [7]. These findings suggest that PM_2.5_ can be a significant source of chronic heavy metal exposure for humans. 

Extensive research has been undertaken to elucidate the health impacts of not only particulate matter itself but also the metals it carries. Pardo et al. [8] reported that mice repeatedly exposed to PM extracts exhibited increased numbers of neutrophils in their bronchoalveolar lavage fluid and elevated levels of cytokines such as TNF-alpha and IL-6 in their lung tissue; this was accompanied by increased reactive oxygen species (ROS) production and oxidative damage to proteins and lipids. These responses were not observed when the mice were exposed to PM extracts with metals removed by chelation. These findings suggest that the metal components of PM play a crucial role in mediating the inflammatory and oxidative damage induced. Similarly, in a panel study of college students, the metallic components of PM_2.5_ were associated with markers of inflammation, such as soluble CD36 and C-reactive protein [9]. It is commonly known that exposure to PM_2.5_ increases inflammatory responses and oxidative damage in humans [10,11,12]. Inhaling PM_2.5_ induces ROS in the body, which can alter the cellular redox status and cause toxicity in humans, even at low levels [13,14,15]. Caused by an imbalance between ROS production and antioxidant defense, oxidative stress can eventually lead to various diseases and aging [16,17]. Our previous research reported that the use of air purifiers to reduce PM_2.5_ reduced oxidative stress in people with CVD [18]. Similarly, Schulz et al. reported that an antioxidant diet can reduce the risk of cardiovascular disease from PM and reduce PM-induced inflammation and oxidative stress [19]. These findings highlight the potential to mitigate the adverse health effects of PM_2.5_ exposure.

Despite the well-documented adverse health effects of PM_2.5_ and the growing understanding of its chemical composition and mechanisms of toxicity, there remains a critical need to elucidate the interactions between PM_2.5_, heavy metal exposure, and oxidative stress in humans. More specifically, understanding how antioxidant intake, such as vitamin supplementation, may modulate these effects is crucial for developing effective preventive strategies. Therefore, the objective of this study was to evaluate the impact of PM_2.5_ and heavy metal exposure on oxidative stress, with a particular focus on how these relationships are influenced by vitamin supplement intake.

## 2. Materials and Methods

### 2.1. Study Participants

This cross-sectional study was conducted among adults aged 20 years and older residing in Cheongju, Chungcheongbuk-do, Republic of Korea, located in the central part of the Korean Peninsula. Current smokers were excluded from the study in order to assess the impact of personal exposure to particulate matter. In total, 114 non-smokers participated in the study, all of whom understood the purpose of the research and gave their voluntary consent. A face-to-face interview survey was conducted, including questions on smoking history, hypertension, diabetes, and dyslipidemia based on physician diagnoses. We also asked whether the participant had taken regular vitamin supplements in the last month. Biological markers were analyzed by collecting venous blood and spot urine samples at the end of the fine dust personal exposure assessment period. Informed consent was obtained from all participants, and this study was reviewed and approved by the Institutional Review Board at Chungbuk National University (CBNU-202005-BMSBBR-0069-01).

### 2.2. Particulate Matter Assessment

To assess personal exposure to particulate matter, we utilized a portable particulate matter sensor, AirBeam (HabitatMap, Brooklyn, NY, USA), the performance of which has been validated by the U.S. Environmental Protection Agency using light scattering technology [20,21]. All study participants carried the AirBeam2 device for 24 h, and PM_2.5_ concentrations were collected at 1-minute intervals. Before the measurements were started, all devices were performance-checked to ensure that the sensors worked correctly at a constant temperature and humidity. The performance tests were conducted in a desiccator chamber, and the accuracy and stability of the instruments were evaluated by ensuring that the measured particulate matter concentration remained constant. Only devices with a coefficient of variation (CV) less than 10% between devices, and within each device’s measurements, were used for the actual measurements. The obtained personal PM_2.5_ data were checked for missing values and outliers, and the final cleaned data were used to calculate the daily personal PM_2.5_ exposure level. Ambient PM_2.5_ concentrations were obtained from the particulate matter monitoring network nearest to the participant’s residence; these were downloaded from the AirKorea website, which is operated by the Ministry of Environment [22]. Data for the same time period as the personal PM_2.5_ assessment were collected for each individual.

### 2.3. Biomarker Measurements for Assessing Metal Exposure

The concentrations of lead and cadmium in blood, and those of chromium and nickel in urine, were determined using a flameless atomic absorption spectrophotometry Ion-Coupled Plasma Mass Spectrometry instrument (Agilent, 7800 series, Santa Clara, CA, USA). For blood lead analysis, samples were pre-treated with 0.2% HNO_3_ and diluent (0.2% NH_4_H_2_PO_4_ in 0.2% Triton X-100); for blood cadmium, the pre-treatment involved 0.2% HNO_3_ and diluent (2.5% NH_4_H_2_PO_4_, 0.15% Mg(NO_3_)_2_ in 0.1% Triton X-100). These were then analyzed using an Atomic Absorption Spectrometer Graphite Furnace (Thermo Fisher, iCE-3000 series, Waltham, MA, USA). For urinary chromium, pre-treatment was carried out with 5% HNO_3_, and for urinary nickel, 2% HNO_3_ was used. The limits of detection (LOD) were 0.12 µg/dL, 0.1 µg/L, 0.15 µg/L, and 0.32 µg/L for lead and cadmium in blood and chromium and nickel in urine, respectively. For samples with concentrations of metal below the LOD, the concentration was replaced by the value of the limit of detection divided by the square root of 2.

### 2.4. Measuring Urinary Total Antioxidant Capacity and Oxidative Stress Markers

The urinary total antioxidant capacity (TAC) was assessed using the copper (Cu) (II) reduction assay with bathocuproinedisulfonic acid disodium salt as the chelating agent (CUPRAC–BCS assay). The results were adjusted to the relative contribution of uric acid (UA) following methods from previous studies [23,24]. To evaluate oxidative stress, urinary malondialdehyde (MDA) and 8-hydroxydeoxyguanosine (8-OHdG) levels were measured. The MDA concentration in urine was quantified by assessing thiobarbituric acid-reactive substances (TBARS) using a high-performance liquid chromatographic system equipped with a fluorescence detector [25]. The LOD of urinary TBARS was 0.07 nM/mL, and the intra-assay CVfor pooled urine samples was 5.25%. The value of the LOD divided by the square root of 2 was substituted in for measurement concentrations below the LOD. The concentration of 8-OHdG was determined using a competitive ELISA kit (8-OHdG check, Japan Institute for the Control of Aging, Kyoto, Japan) with an LOD of 0.19 ng/mL, and the intra-assay CV was 4.18%. All study data fell within the manufacturer’s specified quantification range of 0.5 to 200 ng/mL.

### 2.5. Statistical Analysis

Since the distribution was right-skewed, all data on the levels of biomarkers of exposure to metals and oxidative stress were log-transformed. Differences in the personal PM_2.5_ and the levels of biomarkers, by demographic or lifestyle factors, were compared using Student’s *t*-test. Multiple linear models were used to test the associations between the particulate matter exposure and the levels of exposure biomarkers, as well as the associations between personal particulate matter exposure, biomarkers of exposure to metals, and urinary oxidative stress marker levels. These models included age, sex, body mass index, smoking history, and chronic underlying diseases as covariates. Finally, stratified analyses were performed to compare the association of oxidative stress with exposure factors according to vitamin supplementation status, and interaction terms were included in the model to assess the interaction between these two factors. The level of statistical significance was set at *p* < 0.05. All the statistical analyses were performed using the Statistical Package for the Social Sciences (SPSS) software version 24.0 (IBM, Armonk, NY, USA).

## 3. Results

Table 1 presents the general characteristics of the 114 study participants. The average age of the participants was 61.5 years, with 44.7% being 65 years or older. Women comprised 71.1% of the participants; this may be attributed to the study’s restriction to non-smokers, as smoking prevalence is typically higher among men. All the participants were non-smokers at the time of the study, but 29.0% had a history of smoking, with an average quit duration of 1.9 years before the study. Additionally, 61.4% of participants reported having at least one chronic underlying condition, such as hypertension, diabetes, or dyslipidemia. The average ambient PM_2.5_ concentration on the day of participation was 30.5 µg/m^3^, while the personal PM_2.5_ concentration was 16.9 µg/m^3^. The concentrations of chromium and nickel in urine were 0.52 µg/g creatinine and 5.60 µg/g creatinine, respectively. The total antioxidant capacity measured in urine was 1.98 mM UA equiv./mM creatinine, and the oxidative stress indicators were MDA at 4.60 µg/g creatinine and 8-OHdG at 6.91 µg/g creatinine.

Table 2 presents the concentrations of personal PM_2.5_ exposure, blood and urine heavy metals, and oxidative stress indicators according to participant characteristics. The urinary chromium and nickel concentrations were higher in participants aged 65 and older compared to those under 65, and blood cadmium levels were higher in women than in men (*p* < 0.01). Participants with chronic underlying conditions had significantly higher concentrations of urinary chromium, nickel, and oxidative stress indicators than did those without. There was no significant difference in personal PM_2.5_ exposure levels according to participant characteristics, and no indicators differed based on obesity or vitamin supplement intake.

Table 3 shows the results of the analysis of the association between ambient or personal PM_2.5_ exposure, concentrations of heavy metals, and the total antioxidant capacity in biological samples. The concentration of heavy metals in blood and urine tended to increase with rising individual or ambient PM_2.5_ exposure levels, but this increase was not statistically significant. PM_2.5_ exposure had a non-significant negative association with the total antioxidant capacity in urine (Table 3).

Table 4 evaluates the effects of PM_2.5_ exposure and biomarkers of heavy metal exposure on oxidative stress indicators. When adjusted for age, gender, body mass index, smoking history, and the presence of chronic underlying conditions, blood cadmium and lead concentrations were found to significantly influence urinary MDA concentrations. Urinary chromium and nickel concentrations were also significantly associated with urinary MDA levels. However, personal and ambient PM_2.5_ levels showed no significant association with urinary MDA levels. Personal PM_2.5_ exposure and biomarkers of metal exposure did not show any significant association with the urinary 8-OHdG concentration, although an increase in ambient PM_2.5_ levels tended to marginally increase urinary 8-OHdG levels.

**Figure 1:**
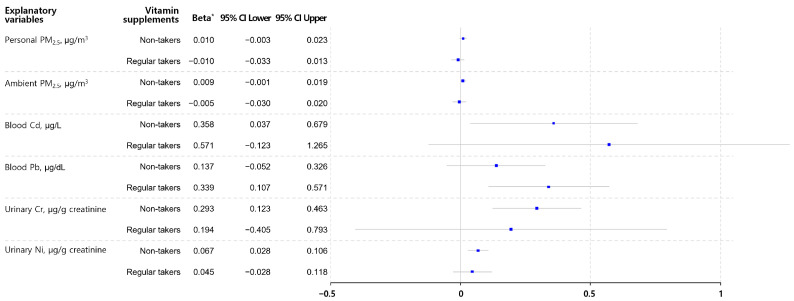
Associations between PM_2.5_ exposure and biomarkers of metals and log-transformed urinary malondialdehyde according to vitamin supplement intake. PM, particulate matter; Cd, cadmium; Pb, lead; Cr, chromium; Ni, nickel; MDA, malondialdehyde. * The beta represents the estimated coefficient of each independent variable in the multiple linear regression model for urinary MDA. The models included covariates such as age, sex, body mass index, smoking history, and chronic underlying diseases.

Figure 1 analyzes the effects of PM_2.5_ exposure and biomarkers of heavy metal exposure on urinary MDA levels based on regular vitamin supplement intake. In the non-vitamin-supplement group, increases in blood cadmium, urinary chromium, and urinary nickel levels were significantly associated with higher urinary MDA concentrations. However, this trend was not observed in the regular vitamin supplement group. For blood lead, a significant association with the urinary MDA concentration was observed only in the regular vitamin supplement group. Nevertheless, the interaction between exposure indicators and vitamin intake was not statistically significant (*p* > 0.05 for all interactions).

## 4. Discussion

Our study evaluated the effects of PM_2.5_ exposure and heavy metal concentrations on oxidative stress within a population of 114 non-smoking adults. The main findings demonstrate that, while personal and ambient PM_2.5_ levels were not directly correlated with elevated heavy metal or oxidative stress markers, significant associations were identified between blood concentrations of cadmium and lead, as well as urinary levels of chromium and nickel, and the oxidative stress marker urinary MDA. Furthermore, this study suggests the potentially moderating role of regular vitamin supplementation in mitigating the oxidative stress induced by heavy metal exposure, pointing to its protective benefits against the harmful impacts of PM_2.5_ and heavy metals.

Fine particulate matter contains various types of metals [7], and exposure to fine dust is expected to increase the body’s heavy metal burden. Indeed, Hu et al. (2021) reported that the concentrations of chromium, nickel, and lead in urine were higher in low- and high-exposure groups compared to the PM non-exposure group [26]. Similarly, Cauci et al. (2022) also reported increases in toxic metals such as cadmium, nickel, and lead in urine following exposure to fine particulate matter [27]. However, our study did not find a correlation between personal exposure levels of PM_2.5_ and the concentrations of heavy metals in blood or urine. This difference may primarily be due to variations in the exposure levels of PM_2.5_. In our study, the personal PM_2.5_ exposure level was 16.9 µg/m^3^, whereas, in the studies by Hu et al., the PM_2.5_ exposure level in the non-exposure group was 62.6 µg/m^3^ [26], indicating a significant difference. Additionally, variations in the composition of PM_2.5_, which can vary in its heavy metal content depending on regional differences in crustal elements and air pollution sources, could also impact results [28]. Therefore, further studies on the association between heavy metal exposure and oxidative stress through PM_2.5_ should not only quantitatively assess the amount of PM_2.5_ but also qualitatively assess its composition. Additionally, other factors contributing to heavy metal exposure, such as diet and occupation, should be evaluated to provide a more comprehensive understanding of its sources and impacts.

The toxic impacts of metals contained in PM_2.5_, such as cadmium, lead, chromium, and nickel, are well documented. Cadmium interferes with physiological functions, induces genotoxicity, suppresses the immune system, and triggers oxidative stress, with a particularly long biological half-life in the kidneys (10 to 30 years) [29,30]. Cadmium is primarily toxic to the kidneys, especially affecting the proximal tubular cells in which it tends to accumulate [29]. Additionally, cadmium can lead to bone demineralization, either directly by damaging bone tissue or indirectly by causing renal dysfunction [29,30]. Lead exposure, especially in high doses during childhood, can cause severe neurological issues; even low-level exposure has been linked to cognitive impairment and elevated blood pressure, with a biological half-life in blood of 28 to 36 days [31,32]. Chromium is a multisystem toxicant affecting the skin, respiratory, gastrointestinal, renal, cardiovascular, hepatic, genetic, hematological, and reproductive systems [33]. Chromium has various toxic properties, such as irritancy and carcinogenicity, along with a related genetic toxicity and allergenicity; it has a urinary half-life of 15 to 41 h, indicating recent exposure [34,35]. Although essential in trace amounts, nickel can cause adverse health effects, including allergic reactions, when exposure is excessive; it has a urinary half-life of 17 to 39 h [36,37]. PM_2.5_ inhalation allows these metals to enter the respiratory system, where they induce oxidative stress and inflammation, leading to a range of health issues [1,7,11]. These metals exert their toxic effects through mechanisms such as inducing oxidative stress, disrupting cellular signaling, and interfering with metal homeostasis, leading to impaired cellular functions and increased oxidative damage [8,36].

This study found no relationship between individual exposure to PM_2.5_ and oxidative stress markers. However, previous research has established a link between the oxidative potential and metal components of PM and oxidative stress [38]. One study by Tan et al. also reported a positive correlation between PM exposure in traffic police officers and urinary 8-OHdG levels [39]. Therefore, when assessing the relationship between oxidative stress and PM exposure, it is important to consider not only the external exposure levels but also the composition and oxidative potential of the particulate matter.

In our study, significant associations were found between the concentrations of cadmium and lead in blood and the concentrations of chromium, nickel, and MDA in urine. This is in line with Hu et al. (2021) [26], who reported significant positive correlations between twelve types of urinary heavy metals, including chromium and nickel, and oxidative stress biomarkers in all participants. A cross-sectional study involving 1907 Koreans also showed a dose-dependent increase in urinary cadmium levels and MDA concentrations [40]. Heavy metals may induce oxidative stress by enhancing cellular redox reactions, generating free radicals, and causing damage to cell membranes, proteins, and DNA [41]. Furthermore, heavy metals can weaken the cellular antioxidant defense system, increasing oxidative stress and lipid peroxidation, which are associated with the decreased activity of antioxidant enzymes like catalase [42].

In this study, significant associations of blood cadmium levels and urinary concentrations of chromium and nickel with the lipid peroxidation marker MDA were observed only in the group that did not take vitamins. This suggests that vitamin intake may mitigate the relationship between heavy metals and oxidative stress. Indeed, vitamins can act as scavengers against reactive oxygen species, including free radicals produced by heavy metals, potentially offering a protective role against metal-related oxidative damage [41].

Animal studies have confirmed that vitamins C and E can reduce oxidative stress caused by cadmium [43]. Additionally, it has been reported that vitamins and the intake of antioxidant-rich foods can prevent cadmium and lead toxicity [44]. These results suggest that antioxidants like vitamins can play a crucial role in alleviating cellular damage related to oxidative stress, effectively reducing the adverse effects of heavy metals from environmental exposure. Therefore, it is recommended that people living in environments with high heavy metal exposure levels should consume sufficient antioxidants.

This study has several limitations. Firstly, due to its small sample size and cross-sectional design, causality is difficult to establish. Furthermore, as the personal exposure levels to PM_2.5_ were only measured over a 24 h period, the long-term effects of exposure could not be assessed. Therefore, there is a need for long-term cohort studies that consider various environmental factors in addition to PM exposure. Additionally, this study was conducted during the COVID-19 epidemic, and mask-wearing might have influenced the actual levels of personal exposure through respiration, potentially altering the exposure patterns compared to those in pre-pandemic times. Secondly, our study participants had an average age of over 60 years, with a high proportion of older adults aged 65. Therefore, it is likely that age-related chronic diseases, such as hypertension, diabetes mellitus, and especially decreased renal function due to age-related kidney disease, affected the urinary biomarkers. This study only examined the presence of comorbidities such as hypertension, diabetes, and dyslipidemia and did not account for kidney disease, which may affect urinary biomarker concentrations. However, we performed statistical analyses that controlled for the effects of age and any confounding comorbidities. Lastly, our study did not differentiate between the types of vitamins participants took, as vitamin intake was broadly categorized to assess overall antioxidant effects. This limitation may obscure the individual impacts of different vitamins on oxidative stress and heavy metal exposure. Although this study confirms the benefits of regular vitamin intake, it is important to specify the different types of vitamins. Future studies should identify specific vitamins to better understand their distinct effects. Despite these limitations, by analyzing various biological markers, this study evaluated the impact of PM_2.5_ exposure and antioxidant interactions on oxidative stress in a Korean population. These findings can be used as foundational data for developing guidelines to minimize health damage caused by PM exposure.

## 5. Conclusions

Our study evaluated the effects of PM_2.5_ exposure and heavy metal concentrations on oxidative stress in a non-smoking adult population. While personal and ambient PM_2.5_ levels did not directly correlate with elevated heavy metal or oxidative stress markers, significant associations were found between blood concentrations of cadmium and lead, as well as urinary levels of chromium and nickel, and the oxidative stress marker, urinary MDA. We also suggest a potentially moderating role of regular vitamin supplementation in mitigating oxidative stress induced by heavy metal exposure. These findings underscore the importance of considering both pollutant composition and antioxidant intake in assessing health risks and developing preventive strategies.

## Figures and Tables

**Table 1:** Study participants’ demographic factors, particulate matter exposure, and biomarkers for metal exposure and oxidative stress.

Variables	Mean (SD) or N (%)
Number	114
Age, years	61.5 (14.6)
Elderly, >65 years	51 (44.7)
Sex, female	81 (71.1)
Body mass index, kg/m^2^	25.4 (3.2)
Overweight or obese, >25 kg/m^2^	62 (54.4)
Ex-smoker	33 (29.0)
Regular vitamin supplement taker *	27 (32.4)
Chronic underlying disease, diagnosed	70 (61.4)
Hypertension	42 (36.8)
Diabetes	27 (23.7)
Dyslipidemia	21 (18.4)
Particulate matter exposure	
Personal PM_2.5_, µg/m^3^	16.9 (11.6)
Ambient PM_2.5_, µg/m^3^	30.5 (15.2)
Biomarkers for exposure and oxidative stress ^†^	
Blood Cd, µg/L	0.97 (1.69)
Blood Pb, µg/dL	0.85 (2.25)
Urinary Cr, µg/g creatinine	0.52 (2.14)
Urinary Ni, µg/g creatinine	5.60 (1.92)
Urinary TAC, mM UA equiv./mM creatinine	1.98 (2.21)
Urinary MDA, µg/g creatinine	4.60 (1.83)
Urinary 8-OHdG, µg/g creatinine	6.91 (1.62)

PM, particulate matter; Cd, cadmium; Pb, lead; Cr, chromium; Ni, nickel; TAC, total antioxidant capacity; MDA, malondialdehyde; 8-OHdG, 8-hydroxydeoxyguanosine. * Individuals who regularly take either a multivitamin or individual vitamin. ^†^ Data are presented as geometric mean values (geometric standard deviations).

**Table 2:** Means of PM exposure and biomarkers of metal and oxidative stress according to study participants’ characteristics.

Variables		Personal PM_2.5_ (µg/m^3^)	Blood Cd (µg/L)	Blood Pb (µg/dL)	Urinary Cr (µg/g creat.)	Urinary Ni (µg/g creat.)	Urinary TAC (mM UA equiv./mM creat.)	Urinary MDA (µg/g creat.)	Urinary 8-OHdG (µg/g creat.)
Age	<65	17.16 (12.07)	0.92 (1.79)	0.97 (2.12)	0.42 (1.92)	4.72 (1.93)	1.87 (2.34)	4.25 (1.95)	6.61 (1.48)
	≥65	16.48 (11.12)	1.06 (1.53)	0.70 (2.38)	0.71 (2.28)	7.25 (1.77)	2.15 (2.05)	5.08 (1.64)	7.31 (1.77)
*p*-value		0.758	0.222	0.074	0.002	0.003	0.354	0.106	0.292
Sex	Male	15.73 (10.94)	0.77 (1.57)	0.94 (1.96)	0.45 (2.19)	4.36 (2.06)	1.77 (2.23)	5.12 (2.00)	6.94 (1.42)
	Female	17.32 (11.91)	1.08 (1.70)	0.81 (2.39)	0.56 (2.12)	6.32 (1.80)	2.08 (2.21)	4.41 (1.75)	6.91 (1.69)
*p*-value		0.509	0.006	0.451	0.217	0.014	0.336	0.233	0.960
BMI	<25	15.78 (11.96)	0.95 (1.67)	0.89 (2.23)	0.52 (2.18)	5.71 (2.02)	2.02 (2.19)	4.3 (1.69)	7.19 (1.56)
	≥25	17.76 (11.33)	0.99 (1.72)	0.82 (2.28)	0.52 (2.13)	5.50 (1.85)	1.95 (2.24)	4.88 (1.94)	6.69 (1.66)
*p*-value		0.367	0.685	0.651	0.982	0.800	0.821	0.266	0.427
Smoking	Never	17.24 (11.6)	1.01 (1.73)	0.81 (2.24)	0.53 (2.18)	5.73 (1.95)	2.05 (2.09)	4.60 (1.87)	6.70 (1.65)
	Ever	15.91 (11.75)	0.84 (1.51)	1.00 (2.29)	0.47 (2.02)	5.11 (1.83)	1.84 (2.53)	4.61 (1.74)	7.46 (1.54)
*p*-value		0.580	0.193	0.338	0.571	0.525	0.520	0.993	0.278
Chronic underlying disease	No	15.83 (12.14)	0.89 (1.80)	0.83 (2.21)	0.44 (1.86)	4.84 (1.86)	1.94 (2.33)	3.87 (1.75)	6.06 (1.63)
	Yes	17.50 (11.30)	1.07 (1.54)	0.86 (2.32)	0.63 (2.37)	6.60 (1.93)	2.02 (2.15)	5.14 (1.83)	7.53 (1.58)
*p*-value		0.456	0.099	0.844	0.030	0.030	0.787	0.014	0.018
Vitamin supplements	Non-taker	17.61 (12.49)	0.97 (1.70)	0.88 (2.22)	0.52 (2.25)	5.47 (1.96)	1.94 (2.20)	4.65 (1.87)	6.79 (1.57)
	Regular taker	15.29 (9.48)	0.98 (1.68)	0.78 (2.34)	0.53 (1.91)	5.91 (1.85)	2.08 (2.26)	4.51 (1.74)	7.18 (1.72)
*p*-value		0.318	0.918	0.520	0.919	0.623	0.666	0.801	0.566

PM, particulate matter; Cd, cadmium; Pb, lead; Cr, chromium; Ni, nickel; TAC, total antioxidant capacity; MDA, malondialdehyde; 8-OHdG, 8-hydroxydeoxyguanosine. Personal PM_2.5_ is presented as the mean (standard deviation), and the others are presented as geometric means with geometric standard deviations.

**Table 3:** Associations between PM exposure and biomarkers of metal exposure and TAC in study participants.

Dependent Variables *	Personal PM_2.5_		Ambient PM_2.5_	
	Beta ^†^	*p*-Value	Beta ^†^	*p*-Value
Blood Cd, µg/L	0.004	0.388	0.001	0.933
Blood Pb, µg/dL	0.005	0.505	0.009	0.226
Urinary Cr, µg/g creatinine	0.006	0.377	0.004	0.505
Urinary Ni, µg/g creatinine	0.007	0.228	0.004	0.368
Urinary TAC, mM UA equiv./mM creatinine	−0.005	0.493	−0.003	0.535

PM, particulate matter; Cd, cadmium; Pb, lead; Cr, chromium; Ni, nickel; TAC, total antioxidant capacity; MDA, malondialdehyde; 8-OHdG, 8-hydroxydeoxyguanosine. * Dependent variables were log-transformed. ^†^ The beta value represents the estimated coefficient of personal or ambient PM_2.5_ in the multiple linear regression models for each dependent variable. The models included covariates such as age, sex, body mass index, smoking history, and chronic underlying diseases.

**Table 4:** Associations between biomarkers of metal exposure and oxidative stress in study participants.

Explanatory Variables	Model for MDA *		Model for 8-OHdG *	
	Beta ^†^	*p*-Value	Beta ^†^	*p*-Value
Personal PM_2.5_, µg/m^3^	0.005	0.414	0.002	0.542
Ambient PM_2.5_, µg/m^3^	0.006	0.246	0.006	0.053
Blood Cd, µg/L	0.412	0.006	−0.001	0.993
Blood Pb, µg/L	0.194	0.011	0.013	0.843
Urinary Cr, µg/g creatinine	0.282	0.001	0.080	0.275
Urinary Ni, µg/g creatinine	0.064	<0.001	0.003	0.818
Urinary TAC, mM UA equiv./mM creatinine	0.025	0.495	0.015	0.539

PM, particulate matter; Cd, cadmium; Pb, lead; Cr, chromium; Ni, nickel; TAC, total antioxidant capacity; MDA, malondialdehyde; 8-OHdG, 8-hydroxydeoxyguanosine. * Dependent variables were log-transformed. ^†^ The beta value represents the estimated coefficient of each explanatory variable in the multiple linear regression model for urinary MDA or 8-OHdG. The models included covariates such as age, sex, body mass index, smoking history, and chronic underlying diseases.

## Data Availability

The data are available from the corresponding author upon reasonable request. The data are not publicly available due to privacy or ethical restrictions.

## References

[1] Kim K.H., Kabir E., Kabir S. (2015). A review on the human health impact of airborne particulate matter. Environ. Int..

[2] Abdullahi K.L., Delgado-Saborit J.M., Harrison R.M. (2013). Emissions and indoor concentrations of particulate matter and its specific chemical components from cooking: A review. Atmos. Environ..

[3] Fuller R., Landrigan P.J., Balakrishnan K., Bathan G., Bose-O’Reilly S., Brauer M., Caravanos J., Chiles T., Cohen A., Corra L. (2022). Pollution and health: A progress update. Lancet Health.

[4] Loomis D., Grosse Y., Lauby-Secretan B., El Ghissassi F., Bouvard V., Benbrahim-Tallaa L., Guha N., Baan R., Mattock H., Straif K. (2013). The carcinogenicity of outdoor air pollution. Lancet Oncol..

[5] Thomaidis N.S., Bakeas E.B., Siskos P.A. (2003). Characterization of lead, cadmium, arsenic and nickel in PM(2.5) particles in the Athens atmosphere, Greece. Chemosphere.

[6] Choi E., Yi S.-M., Lee Y.S., Jo H., Baek S.-O., Heo J.-B. (2022). Sources of airborne particulate matter-bound metals and spatial-seasonal variability of health risk potentials in four large cities, South Korea. Environ. Sci. Pollut. Res. Int..

[7] Liu Y., Li S., Sun C., Qi M., Yu X., Zhao W., Li X. (2018). Pollution Level and Health Risk Assessment of PM-Bound Metals in Baoding City Before and After the Heating Period. Int. J. Environ. Res. Public Health.

[8] Pardo M., Porat Z., Rudich A., Schauer J.J., Rudich Y. (2016). Repeated exposures to roadside particulate matter extracts suppresses pulmonary defense mechanisms, resulting in lipid and protein oxidative damage. Environ. Pollut..

[9] Zhang L., Fang B., Wang H., Zeng H., Wang N., Wang M., Wang X., Hao Y., Wang Q., Yang W. (2023). The role of systemic inflammation and oxidative stress in the association of particulate air pollution metal content and early cardiovascular damage: A panel study in healthy college students. Environ. Pollut..

[10] Turner M.C., Andersen Z.J., Baccarelli A., Diver W.R., Gapstur S.M., Pope CA 3rd Prada D., Samet J., Thurston G., Cohen A. (2020). Outdoor air pollution and cancer: An overview of the current evidence and public health recommendations. CA Cancer J. Clin..

[11] Lee C.W., Vo T.T.T., Wu C.Z., Chi M.C., Lin C.M., Fang M.L., Lee I.T. (2020). The Inducible Role of Ambient Particulate Matter in Cancer Progression via Oxidative Stress-Mediated Reactive Oxygen Species Pathways: A Recent Perception. Cancers.

[12] Li Z., Liu Q., Xu Z., Guo X., Wu S. (2020). Association between short-term exposure to ambient particulate air pollution and biomarkers of oxidative stress: A meta-analysis. Environ. Res..

[13] Park J., Park E.H., Schauer J.J., Yi S.M., Heo J. (2018). Reactive oxygen species (ROS) activity of ambient fine particles (PM(2.5)) measured in Seoul, Korea. Environ. Int..

[14] Wang Y., Li Y., Gao Y., Kang J., Wang W., Yong Y.L., Qu X., Dang X., Shang D., Shao Y. (2023). Fine particulate matter exposure disturbs autophagy, redox balance and mitochondrial homeostasis via JNK activation to inhibit proliferation and promote EMT in human alveolar epithelial A549 cells. Ecotoxicol. Environ. Saf..

[15] Wyatt L.H., Devlin R.B., Rappold A.G., Case M.W., Diaz-Sanchez D. (2020). Low levels of fine particulate matter increase vascular damage and reduce pulmonary function in young healthy adults. Part. Fibre Toxicol..

[16] Pizzino G., Irrera N., Cucinotta M., Pallio G., Mannino F., Arcoraci V., Squadrito F., Altavilla D., Bitto A. (2017). Oxidative Stress: Harms and Benefits for Human Health. Oxid. Med. Cell. Longev..

[17] Sharifi-Rad M., Anil Kumar N.V., Zucca P., Varoni E.M., Dini L., Panzarini E., Rajkovic J., Tsouh Fokou P.V., Azzini E., Peluso I. (2020). Lifestyle, Oxidative Stress, and Antioxidants: Back and Forth in the Pathophysiology of Chronic Diseases. Front. Physiol..

[18] Eom S.Y., Kim A., Lee J.H., Kim S.M., Lee S.Y., Hwang K.K., Lim H.J., Cho M.C., Kim Y.D., Bae J.W. (2022). Positive Effect of Air Purifier Intervention on Baroreflex Sensitivity and Biomarkers of Oxidative Stress in Patients with Coronary Artery Disease: A Randomized Crossover Intervention Trial. Int. J. Environ. Res. Public Health.

[19] Schulz A.J., Mentz G.B., Sampson N.R., Dvonch J.T., Reyes A.G., Izumi B. (2015). Effects of particulate matter and antioxidant dietary intake on blood pressure. Am. J. Public Health.

[20] Jiao W., Hagler G., Williams R., Sharpe R., Brown R., Garver D., Judge R., Caudill M., Rickard J., Davis M. (2016). Community Air Sensor Network (CAIRSENSE) project: Evaluation of low-cost sensor performance in a suburban environment in the southeastern United States. Atmos. Meas. Tech..

[21] Mukherjee A., Brown S.G., McCarthy M.C., Pavlovic N.R., Stanton L.G., Snyder J.L., D’Andrea S., Hafner H.R. (2019). Measuring Spatial and Temporal PM(2.5) Variations in Sacramento, California, Communities Using a Network of Low-Cost. Sensors.

[22] Environment T.M.O. Annual Report of Ambient Air Quality in Korea. https://www.airkorea.or.kr/eng.

[23] Campos C., Guzmán R., López-Fernández E., Casado A. (2010). Urinary uric acid and antioxidant capacity in children and adults with Down syndrome. Clin. Biochem..

[24] Campos C., Guzmán R., López-Fernández E., Casado A. (2009). Evaluation of the copper(II) reduction assay using bathocuproinedisulfonic acid disodium salt for the total antioxidant capacity assessment: The CUPRAC-BCS assay. Anal. Biochem..

[25] Agarwal R., Chase S.D. (2002). Rapid, fluorimetric-liquid chromatographic determination of malondialdehyde in biological samples. J. Chromatogr. B.

[26] Hu W., Wang Y., Wang T., Ji Q., Jia Q., Meng T., Ma S., Zhang Z., Li Y., Chen R. (2021). Ambient particulate matter compositions and increased oxidative stress: Exposure-response analysis among high-level exposed population. Environ.Int..

[27] Cauci S., Tavano M., Curcio F., Francescato M. (2022). Biomonitoring of urinary metals in athletes according to particulate matter air pollution before and after exercise. Environ. Sci. Pollut. Res..

[28] Kundu S., Stone E.A. (2014). Composition and sources of fine particulate matter across urban and rural sites in the Midwestern United States. Environ. Sci. Process Impacts.

[29] Bernard A. (2008). Cadmium & its adverse effects on human health. Indian J. Med. Res..

[30] Jarup L., Akesson A. (2009). Current status of cadmium as an environmental health problem. Toxicol. Appl. Pharmacol..

[31] Sule K., Umbsaar J., Prenner E.J. (2020). Mechanisms of Co, Ni, and Mn toxicity: From exposure and homeostasis to their interactions with and impact on lipids and biomembranes. Biochim. Et Biophys. Acta (BBA) Biomembr..

[32] Patrick L. (2006). Lead toxicity, a review of the literature. Part 1: Exposure, evaluation, and treatment. Altern Med. Rev..

[33] Pavesi T., Moreira J.C. (2020). Mechanisms and individuality in chromium toxicity in humans. J. Appl. Toxicol..

[34] Dayan A.D., Paine A.J. (2001). Mechanisms of chromium toxicity, carcinogenicity and allergenicity: Review of the literature from 1985 to 2000. Hum. Exp. Toxicol..

[35] Kerger B.D., Paustenbach D.J., Corbett G.E., Finley B.L. (1996). Absorption and elimination of trivalent and hexavalent chromium in humans following ingestion of a bolus dose in drinking water. Toxicol. Appl. Pharmacol..

[36] Nordberg G.F., Fowler B.A., Nordberg M. Handbook on the Toxicology of Metals. Academic Press: Cambridge, MA, USA, 2014.

[37] Dooyema C.A., Neri A., Lo Y.C., Durant J., Dargan P.I., Swarthout T., Biya O., Gidado S.O., Haladu S., Sani-Gwarzo N. (2012). Outbreak of fatal childhood lead poisoning related to artisanal gold mining in northwestern Nigeria, 2010. Environ. Health Perspect..

[38] Liu L., Urch B., Szyszkowicz M., Evans G., Speck M., Van Huang A., Leingartner K., Shutt R.H., Pelletier G., Gold D.R. (2018). Metals and oxidative potential in urban particulate matter influence systemic inflammatory and neural biomarkers: A controlled exposure study. Environ.Int..

[39] Tan C., Lu S., Wang Y., Zhu Y., Shi T., Lin M., Deng Z., Wang Z., Song N., Li S. (2017). Long-term exposure to high air pollution induces cumulative DNA damages in traffic policemen. Sci. Total Environ..

[40] Eom S.Y., Seo M.N., Lee Y.S., Park K.S., Hong Y.S., Sohn S.J., Kim Y.D., Choi B.S., Lim J.A., Kwon H.J. (2017). Low-Level Environmental Cadmium Exposure Induces Kidney Tubule Damage in the General Population of Korean Adults. Arch. Environ. Contam. Toxicol..

[41] Valko M., Morris H., Cronin M.T. (2005). Metals, toxicity and oxidative stress. Curr. Med. Chem..

[42] Vlahogianni T.H., Valavanidis A. (2007). Heavy-metal effects on lipid peroxidation and antioxidant defence enzymes in mussels. Chem. Ecol..

[43] Sen Gupta R., Sen Gupta E., Dhakal B.K., Thakur A.R., Ahnn J. (2004). Vitamin C and vitamin E protect the rat testes from cadmium-induced reactive oxygen species. Mol. Cells.

[44] Zhai Q., Narbad A., Chen W. (2015). Dietary strategies for the treatment of cadmium and lead toxicity. Nutrients.

